# The impact of the loss of first permanent molars on the duration of treatment in patients treated with orthodontic space closure and without skeletal anchorage

**DOI:** 10.1186/s40510-022-00427-2

**Published:** 2022-09-12

**Authors:** Paula Coutinho Cardoso, Paulo Mecenas, David Normando

**Affiliations:** 1 Orthodontist at Private, Belém, Pará Brazil; 2 Orthodontic Department, University Center FIBRA, Belém, Pará Brazil; 3 Faculty of Dentistry, Federal University of Pará (UFPA), Rua Augusto Correa 01, Belém, Pará 66075-110 Brazil

**Keywords:** Orthodontic brackets, Malocclusion, First permanent molar

## Abstract

**Background:**

This study aims to evaluate the impact of the loss of permanent molars on the duration of orthodontic treatment for space closure and without skeletal anchorage.

**Methods:**

Records at the beginning (T0) and the end (T1) of orthodontic treatment were selected retrospectively. Patients were divided into two groups: loss of molar (*n* = 19) and control, without loss (*n* = 24). The impact of loss on treatment time was assessed using multiple linear regression adjusted for the number of absences, bonding failures, age, sex, PAR index at T0 and T1 at *p*<0.05. Treatment time was also evaluated by the number of losses and which arches were involved (upper, lower). The systematic and random errors for the PAR index were verified using the intraclass correlation coefficient (ICC) and the Dahlberg formula, respectively.

**Results:**

A small random error (1.51) and excellent replicability (ICC = 99.6) were observed. Overall average treatment time was 22.5 months (± 7.95) for the group without loss and 44.7 months (± 17.3) with a loss. Treatment time was longer in cases where there was a higher number of missing molars and when both arches were involved. In addition to the loss (*β* = 4.25, *p* < 0.001), the number of missed appointments (*β* = 2.88, *p* < 0.001) had a significant effect and increased treatment time. Bonding failures, gender, age, and PAR index at T0 and T1 were not significantly associated with treatment time in the multivariate model (*p* > 0.05).

**Conclusion:**

Loss of the first permanent molar has a negative impact on orthodontic treatment time in cases of space closure. The treatment time is longer when there are more tooth losses and arches involved. Treatment time also increases with greater numbers of missed clinical appointments.

## Background

Loss of the first permanent molar is highly prevalent in low socioeconomic groups [1, 2] as a consequence of the presence of extensive dental carious lesions [3]. However, the extraction of permanent molars shows a high occurrence even in populations with better socioeconomic status, due to their high susceptibility to enamel hypomineralization (11%) [3, 4].

Among the changes in the development of the occlusion resulting from the loss of permanent molars are an inclination of adjacent teeth to the area of loss [1, 4–7], midline deviation [1, 7], distal migration of lower canines with incisor migration [1], lingual inclination of lower incisors [8], periodontal problems [4], and temporomandibular dysfunction [4]. A recent systematic review showed that in cases of loss of the permanent molar, a spontaneous space closure might occur in 45.5% to 85.2% of the cases [9]. This great variability is related to the methodology and the patients’ heterogeneity included in the primary studies. When the space is not completely closed, the need for orthodontic treatment increases. Thus, the decision will fall between closing the remaining space or reopening it for further rehabilitation.

A systematic review [10] showed that the duration of orthodontic treatment in adults does not differ from adolescents. However, this review did not include studies with individuals with loss of the first permanent molar. Progressive occlusal alterations resulting from loss of molars could lead to a longer treatment time in adult patients due to the accumulated needs and the progressive nature of the occlusal changes. Another consequence of loss is alveolar ridge atrophy, which may make orthodontic movement difficult. In patients who required mesiodistal tooth movement in edentulous alveolar ridges, a mean treatment time of 1.54 years until space closure and 1.44 years until space opening was reported [11], suggesting that there is no difference between these two treatment options. However, the impact of these mechanics on the total time of orthodontic treatment has not been evaluated.

Thus, orthodontic treatment in adults with tooth loss could become more complex, leading to longer treatment [12, 13]. However, there appears to be no scientific evidence on this issue. Therefore, this study aims to assess the impact of the loss of the first permanent molar, the most frequently tooth loss [7], on the length of orthodontic treatment in cases where the total closure of the remaining space has been planned.

## Methods

### Study design

This retrospective study followed the STROBE guideline [14] and was submitted and approved by the Ethics and Research Committee of the Center for Biological and Health Sciences of the Federal University of Pará (CAAE: 45587121.1.0000.0018).

### Characteristics

All orthodontic records for patients who had already completed orthodontic treatment and been treated in a private clinic by a single orthodontist were evaluated. All cases required initial and final dental casts. Cases with damaged or incomplete records were excluded.

### Participants

**Fig. 1:**
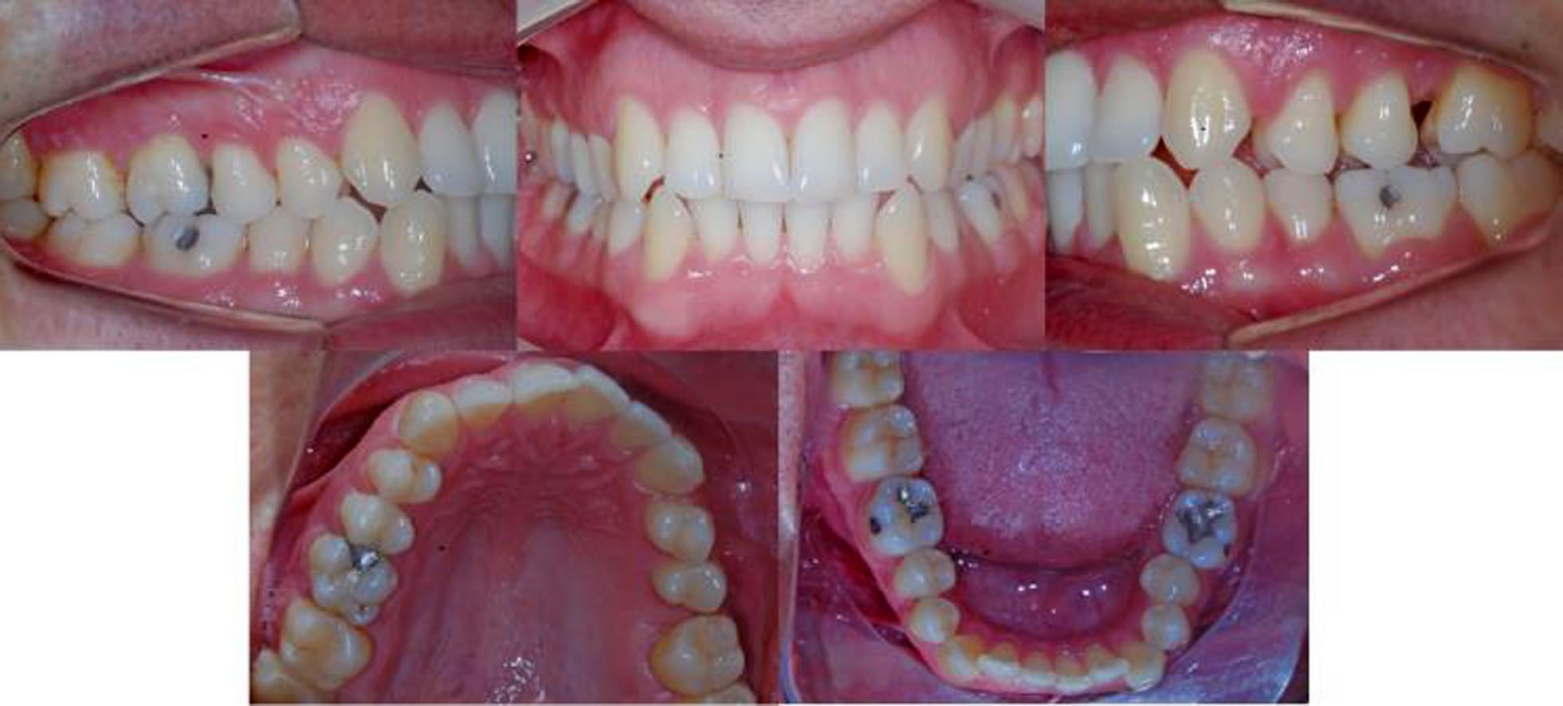
Pretreatment photographs of an adult patient with one permanent first molar loss

Patients were orthodontically treated with pre-adjusted brackets, Roth prescription 0.022″ × 0.028″ slot. The sample was divided into two groups. The first one consisted of patients who had loss of at least one permanent first molar (loss group, Fig. 1), and the second one consisted of individuals without tooth loss (control group). All patients in the loss group were treated with space closure without temporary anchorage devices (TADs). The inclusion criteria were patients in permanent dentition greater than or equal to 14 years, without loss of other dental elements—except for first permanent molar, impacted teeth, or agenesis. Surgical patients with craniofacial syndromes, cleft lips and palate, agenesis, impacted teeth, or patients who had undergone previous orthodontic treatment with fixed appliances, or patients with 10 or more monthly absences during treatment were excluded.

**Fig. 2:**
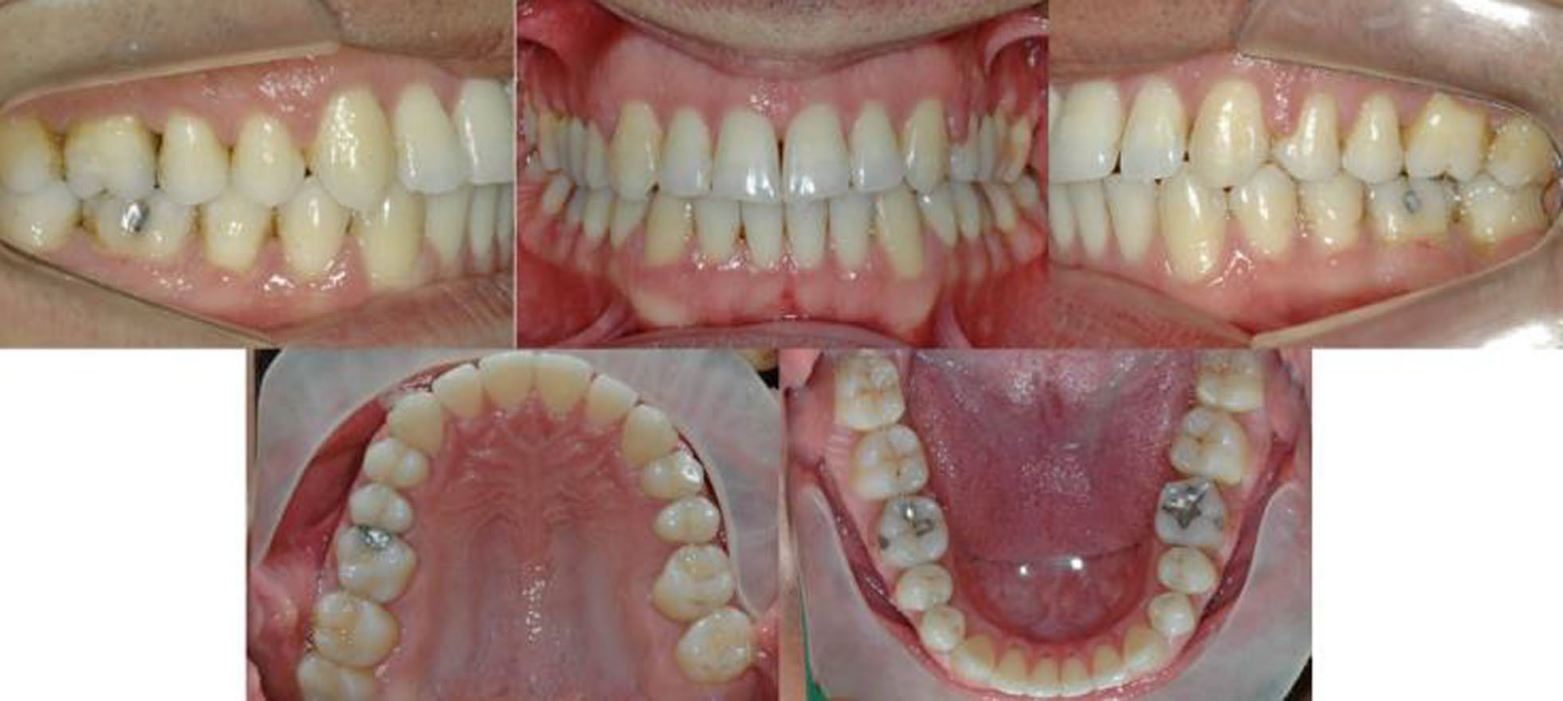
Posttreatment photographs of an adult patient with one permanent first molar loss who as treated with space closure

In this clinical example, patient had loss one first permanent molar on the upper arch, class III, and presented a moderate crowding on the anterior region of both arches. Treatment consisted of correcting these conditions and space closure of the molar loss, and at the end of treatment, all the goals were achieved (Fig. 2). The total treatment time was 20 months, and patients had no missed appointments and only one debonding.

### Variables

Data related to the length of treatment (dependent variable) were collected from the patient's clinical record, and related to the independent variables included, such as the number of tooth losses, sex, age, remaining space, initial angulation of the second permanent molar, number of absences from clinical appointments (months without attendance), bonding failure frequency, Peer Assessment Rating (PAR) index [15], and the sites of losses (maxilla and/or mandible).

### Data and measurements

All dental casts were scanned with the TRIOS® Pod device (3Shape, Copenhagen, Denmark). The severity of malocclusion was evaluated using the initial (T0) and final (T1) PAR index [15], and the remaining space was measured by the initial distance between the mesial marginal ridge of the second permanent molar and the distal marginal ridge of the second premolar. Measurements were obtained by a single calibrated examiner (P.C.) using the 3Shape Ortho Viewer software (Copenhagen, Denmark). Dental angulation was evaluated using panoramic radiography images, and tracings were performed using the method described by Ursi et al. [16]

### Statistical analysis

To analyze the replicability of measurements, all T0 models were reassessed after 30 days. The analysis of systematic and random errors was evaluated using ICC and Dahlberg's formula, respectively.

The association of each independent variable with the dependent variable was verified through the univariate linear regression model. Then, in the multivariate model, only the variables that presented a value of *p* < 0.10 were included.

All statistical analyses were performed using Jamovi software (version 1.6.16, Sydney, Australia).

## Results

### Participants

**Fig. 3:**
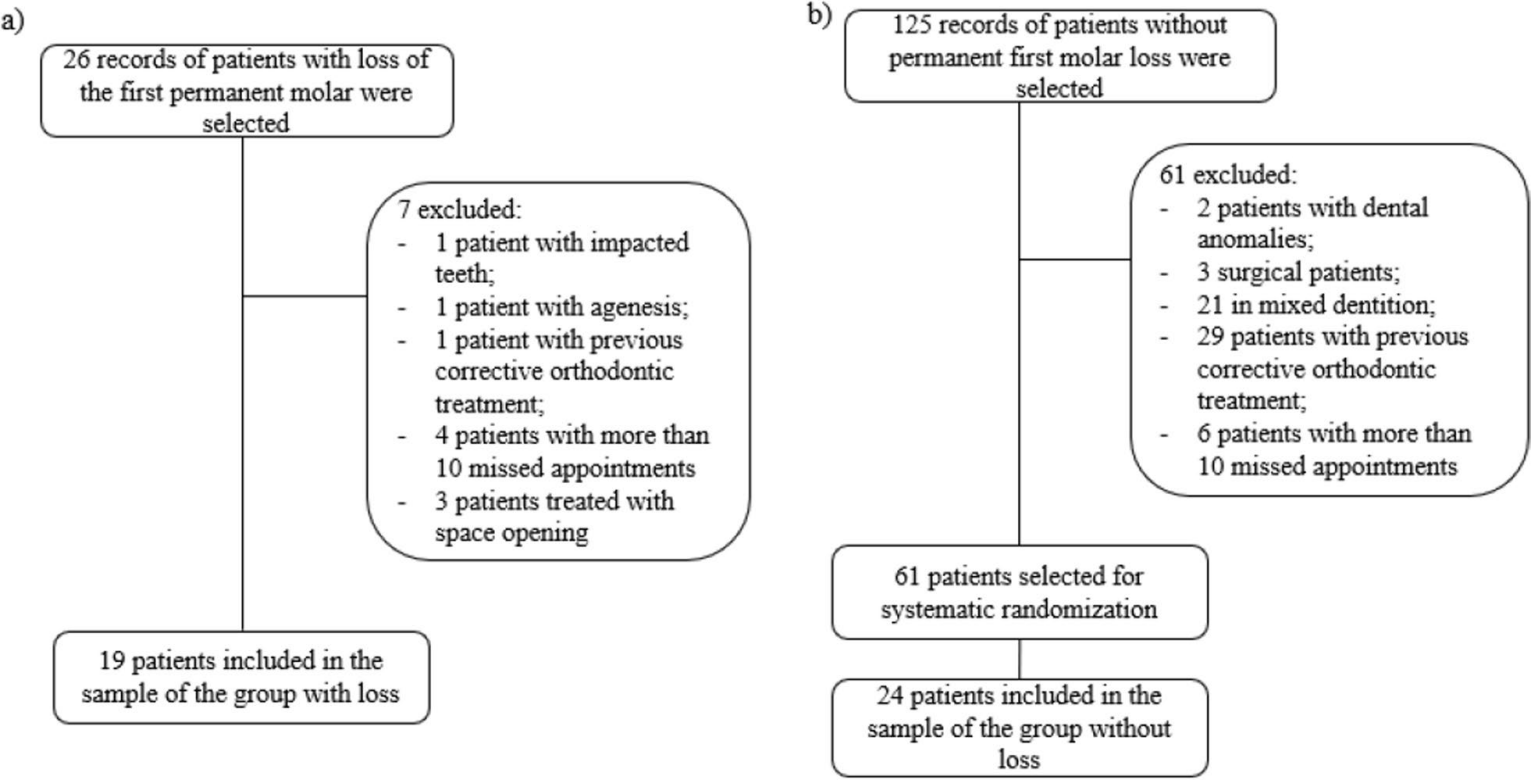
Flow diagram of selected participants for the loss (**a**) and no loss (**b**) group

Initially, 29 medical records of patients who had loss of the first permanent molars were selected from the clinical records. After evaluating their records, three patients were excluded. One had an impacted tooth, and another had agenesis. One patient had previously received fixed corrective orthodontic treatment, and four had more than ten absences during treatment. Another three were treated with space reopening. This group was excluded from the analysis due to its low representativeness. In the end, 19 patients were included in the sample of the group with loss and space closure (Fig. 3a).

For the group without loss, 125 patients were previously selected. Of these, 61 were excluded: 2 patients had dental anomalies, 3 cases were surgical, 21 had incomplete permanent dentition, 29 had previously received corrective orthodontic treatment, and 6 had more than 10 absences. Thus, 64 patients were included in the systematic randomization that selected 24 patients (Fig. 3b).

### Descriptive data

The sample included a total of 43 patients, 30 women (69.7%), and 13 men (30.2%). Nineteen patients (44.2%) had loss of at least one permanent first molar, and 24 patients had no loss (55.8%). Age ranged from 14 to 73 years in the group with loss, and from 14 to 58 in the group without loss.

### Main results

Overall, mean treatment time was 44.7 months (± 17.3) for the group with loss and 22.5 months (± 7.95) for the group without loss. The mean number of absences was 4.42 (± 3.49) in the group with loss and 2.25 (± 2.44) in the group without loss. The mean number of appliance detachments for the group with loss was 3.79 (± 3.65) and 2.25 (± 2.44) for the group without loss. The PAR index at T0 was 21.9 (± 5.94) for the group with loss and 14.8 (± 7.38) for the group without loss, and at T1, 5.63 (± 2.63) and 3.92 (± 3.12) for the groups with and without loss, respectively (Tables 1 and 2).

**Table 1:** Mean and standard deviation (SD) for age at T0, treatment time, number of absences, number of orthodontic appliance breakages, PAR at T0 and T1, % PAR corrected in groups with loss and without loss

Group (n)	Age (± SD)	Treatment time	Absences (f)	Debonding	PAR T0	PAR T1	% PAR
Loss (*n* = 19)	34.9 (± 13.0)	44.7 (± 17.3)	4.42 (± 3.49)	3.79 (± 3.65)	21.9 (± 5.94)	5.63 (± 2.63)	73.5 (± 11.9)
Without Loss (*n* = 24)	23.8 (± 13.1)	22.5 (± 7.95)	2.25 (± 2.44)	2.25 (± 2.91)	14.8 (± 7.38)	3.92 (± 3.12)	70.4 (± 24.2)

**Table 2:** Mean and standard deviation of treatment time according to the number of losses and number of arches involved

		Number of losses				Dental Arches	
	0 (*n* = 24)	1 (*n* = 2)	2 (*n* = 8)	3 (*n* = 6)	4 (*n* = 3)	1 (*n* = 9)	2 (*n* = 10)
Treatment Time	22.5	30.5	36.8	57.2	50.7	36.1	52.5
X ± SD	± 7.95	± 14.8	± 6.78	± 18.5	± 24.5	± 8.45	± 19.9

Two patients (10.5%) had loss of only one permanent first molar, eight (42.1%) had two losses, six (31.6%) had three losses, and three (15.8%) had four losses. The mean treatment time, in months, increased according to the number of losses, being 30.5 (± 14.8) for one loss, 36.8 (± 6.78) for two losses, 57.2 (± 18.5) for three losses, and 50.7 (± 24.5) for the loss of the four first permanent molars. For cases with tooth loss in only one arch (maxilla or mandible *n* = 9), the mean treatment time was 36.1 months (± 8.45), and 52.5 (± 19.9) when in both arches (*n* = 10) (Table 2). Regarding dental classification, 24 patients had a Class I canine relationship, with a mean treatment time of 32.0 months (± 19.8), 15 patients were Class II, with a mean time of 33.1 months (± 13.8), and only four patients were Class III, with a mean treatment time of 31.5 months (± 11.1). The mean of the initial remaining space was 2.18 mm, and the initial angulation was 26.9º.

Regarding systematic error, an ICC of 96% between the two measurements was found (95% CI 0.93–0.98), indicating excellent replicability of the measurements. A small random error was observed, indicating that the mean difference between the two PAR T0 evaluation times was 1.51 (Table 3).

**Table 3:** Systematic error and 95% confidence interval for the intraclass correlation coefficient (ICC) and the random error of the variable PAR T0

Variable	Systematic error, ICC (IC 95%)	Random error, *Dahlberg*
PAR T0	0.96 (0.93–0.98)	1.51

The variable “dental arch” related to the location of the loss, whether only in the upper or lower arch or in both arches, was not included in the multivariate model because it presented high collinearity with the variable number of losses (VIF = 16.17). The independent variables number of losses (*p* < 0.001), number of absences (*p* < 0.001), PAR at T0 (*p* < 0.001), PAR at T1 (*p* = 0.056), sex (*p* = 0.045), age (*p* = 0.013), remaining space (*p* 0.001), and initial angulation (*p* < 0.001) were included in the multivariate model because they presented a value of *p* < 0.1. These variables together explained 77.4% of the variability of treatment time; however, only the number of lost molars (*p* = 0.027) and the number of missed appointments (*p* < 0.001) had a significant association with time in the multivariate model with R2 adjusted equal to 0.774. The assumptions of normality (*p* = 0.4), heteroscedasticity (*p* = 0.45), and multicollinearity (VIF = 1.22) were met. The results of the *β* coefficients showed that for each loss of the first permanent molar, the treatment time increased by 3.78 months on average, and for each absence, the treatment time increased by 2.83 months (Table 4).

**Table 4:** Variables associated with of orthodontic treatment duration (dependent variable) by the univariate and multiple linear regression model

Independent Variables	Univariate model			Multivariate model			
	*p* value	Adjusted *p* value	CI (95%)	*β*	*R*^2^	Adjusted *R*^2^	*F* test
Number of 1st molar losses	< 0.001*	0.027	0.455–7.099	3.777	0.817	0.774	19.0
Months of missed appointments	< 0.001*	< 0.001*	1.918–3.751	2.834			
PAR T0	< 0.001*	0.180	− 0.142 to 0.729	0.293			
PAR T1	0.056*	0.174	− 0.304 to 1.619	0.657			
Sex	0.045*	0.765	− 6.860 to 5.087	− 0.886			
Age at T0	0.013*	0.152	− 0.057 to 0.356	0.140			
Remaining Space	< 0.001*	0.288	− 0.042 to 0.137	0.047			
Angulation	< 0.001*	0.771	− 1.246 to 0.932	0.157			
Dental Arches	< 0.001*						
Number of debondings	0.207						
Previous Orthodontic Treatment	0.457						
Class I, II or III	0.874						

CI, Confidence Interval

^*^Statistically significant at *p* < 0.10

The variables number of deboning, previous orthodontic treatment, and Angle’s classification (class I, II, or III) presented a value of *p* > 0.1 and were not included in the multivariate model because they were not statistically significant.

## Discussion

The results showed that the treatment time is influenced by the number of losses of the first permanent molars and the number of missed appointments during treatment. Together, these variables explain 77.4% of the variability observed in treatment time. Meta-analyses have reported a mean treatment time with a fixed appliance in adults and adolescents ranging from 20.02 months (95% CI 19.71–20.32) [17] to 4.9 months (95% CI 24.6–25.1) [18]. These results agree with the findings obtained in the group without loss in this study, in which an average of 22.5 months was observed. However, patients with loss of the first permanent molar had a longer mean treatment time (44.7 ± 17.3). This information makes a marked contribution to predicting the orthodontic treatment duration, which is extremely important to the patient [17]. Thus, in a patient with loss of first molars, whose treatment proposal will consider the space closure without anchorage devices, it should be noted that the average treatment time will be twice as long, and may present a large variability, explained in part by some factors.

Among the factors that model the variability of treatment time, one is the number of molars lost. Patients who had only one permanent first molar missing had a shorter treatment time when compared to those who had lost two, three, or all their first molars. While the treatment time in patients without a loss was almost two years, patients with loss of one or two molars were treated for approximately 2.5 to 3 years. The treatment time for patients who lost the four first molars was similar (50.7 ± 24.5) to patients who had three losses (57.2 ± 18.5) and much longer than the other patients with losses. However, these means must be viewed with caution as only two patients with one loss and three patients with loss of the four first molars were included.

The number of arches involved was another variable examined, although it was not included in the multivariate model due to the high collinearity with the number of molar losses. For cases with tooth loss in only one arch (maxilla or mandible), the mean treatment time was about three years and just over four years when losses occurred in both arches. The loss of molars in the lower arch causes a greater occurrence of a Class II canine relationship and a lingualization of the incisors [18], especially the lower ones. The treatment of these alterations, which generally involves the use of intermaxillary elastics, makes orthodontic mechanics more complex since it exacerbates a possible undesirable effect on the inclination of the maxillary anterior teeth during space closure.

The loss of the first permanent molars can cause several changes to the occlusion, such as the movement of antagonist and adjacent teeth to the edentulous area, which can lead to occlusal and periodontal problems [19]. In these cases, orthodontists have two treatment options: opening or closing the remaining spaces, and little is known about the duration of orthodontic treatment comparing these cases. In this study, all patients in the loss group (*n* = 19) were treated with space closure and had a mean treatment time of 44.7 months (Fig. 1). A previous study that evaluated treatment time in patients treated with reopening versus closure of edentulous spaces of the mandibular first molar in patients treated with or without miniscrews found a similar mean treatment time for the two procedures. The time for the total space closure was 1.54 years and 1.44 years for the reopening of spaces, indicating that there was no clinical difference between these two therapeutic options. The authors did not report the total treatment time.

In this study, none of the patients included in the sample were treated with skeletal anchorage, which may have contributed to the longer treatment time observed in the group with loss. It is known that TADs can help with different mechanics of orthodontic treatment, such as molar uprighting, and reduced chair time and side effects [20]. Furthermore, the use of TADs seems to be related to a reduction in treatment time in cases of anterior retraction after extraction of the first premolars [21]. Thus, it is possible that the use of these devices may contribute to a shorter treatment time in cases of space closure in the molar area. Studies that assess the effect of skeletal anchorage in reducing the treatment time of patients with molar loss are needed.

Another factor that was related to the increase in treatment time was the number of missed appointments during orthodontic treatment (*p* < 0.001), indicating that with each absence, the treatment time increases by 2.8 months. Previous studies [13, 22, 23] have shown that patient compliance to orthodontic treatment also has a significant influence on treatment duration. However, the results observed in the present study seem to be more decisive than those reported in a previous study that reported the length of treatment in Class III. This may be associated with the fact that patients who miss appointments more often may also be patients who are less cooperative with treatment. This association is in agreement with a previous study [22] that reported that the duration of orthodontic treatment in adults is mainly influenced by factors related to patients’ compliance, such as number of months the case was left with no monitoring due to missed or canceled appointments and number of appliances breakages. Mechanics of closing spaces may depend on professional control and patient cooperation, with the use of intermaxillary elastics, chains, or coils. Thus, in addition to the month lost due to missed appointments, treatment time may be extended due to the lack of activation forces that are necessary to close spaces and complete orthodontic treatment.

In this study, there was an improvement of 73.5% in the PAR index for the group with loss, and 70.4% for the group without loss. For orthodontic treatment to be considered good quality, an average percentage reduction in the PAR index greater than 70% is proposed [24]. The higher percentage of correction in the group with loss may be associated with a greater severity of malocclusion observed in this group at the beginning of treatment (T0). The severity of the initial malocclusion (PAR T0), despite a significant association with the treatment duration in the univariate analysis (*p* 0.01), did not show a significant association in the multivariate analysis (*p* = 0.088) when adjusted to other variables. The final PAR (T1) showed a marginal association in the univariate analysis (*p* = 0.056) which was not significant in the multivariate analysis (*p* = 0.166), a finding supported by results from previous studies [22, 25]. However, other studies [13, 26, 27] have reported that the initial malocclusion index is significantly associated with treatment duration. This difference may be related to the fact that the PAR index does not assess some initial aspects of malocclusion such as root angulation and parallelism [28]. Besides that, occlusal changes measured in the PAR index are dependent on other variables included in the regression model, mainly changes resulting from tooth loss.

Although the number of bonding failures is also a variable associated with patient cooperation, this variable was not related to treatment time (*p* = 0.207) which disagrees with several previous studies [22, 23, 29]. However, the mean number of appliance breakages, both in the patients' group who presented loss of the first molar (3.79 ± 3.65) and in the group without loss (2.25 ± 2.91), was relatively small. Therefore, it presented a small variability to the point of influencing treatment time.

It is reported in the literature that the longer the loss time, the greater the chances that this region will undergo changes such as the mesial inclination of the second molar, distal inclination of the second premolar, bone loss, and alveolar crest remodeling [7, 8]. In addition, space closure in patients who have had recent first molar loss seems to have more predictable results than older losses [5]. This relationship between the time of loss and occlusal alterations was not evaluated in this study. It is difficult to obtain this type of information in a retrospective study where most patients are not able to report, with a reasonable accuracy, the time elapsed between the moment of loss of the first permanent molar and the beginning of treatment.

Other morphological aspects related to the consequences of first permanent molar loss, such as the amount of space remaining and the degree of inclination of adjacent teeth, could contribute to the determination of treatment duration. The remaining space was evaluated through the digitized models, measuring the space between the crowns of the permanent second molar and second premolar. However, this measurement may not represent the real dimension of the space, as there is an inclination of the adjacent teeth toward the edentulous region. Because of this, the initial angulation of the second permanent molars was also evaluated. It is known that the teeth that are closer to the site of loss are the ones that suffer the most significant angular changes [7], and orthodontists can close spaces of 10 mm or more in edentulous areas, even though it is considered a difficult movement to perform and with high rates of relapses [30]. Even so, the degree of inclination of the second permanent molar (60.8 ± 39.8) and the remaining space (4.93 ± 3.8) were not related to the duration of orthodontic treatment.

In this study, patients aged from 14 to 73 years were included and it was observed that the age at the beginning of the treatment had no significant influence on the treatment duration in patients with loss of the first permanent molar. The univariate analysis showed a significant influence of age on treatment time, which lost significance when adjusted for other variables, especially tooth loss and the number of appointment absences. It would be expected that older patients would have a more significant loss of the alveolar bone ridge, making orthodontic movement difficult and possibly causing a longer treatment time. However, it is also possible that the longer the time elapsed since the loss, the greater the spontaneous migration of teeth into the area of loss. Therefore, the data obtained in this study indicated that molar loss, more commonly seen in older individuals, increases the treatment duration regardless of whether it occurs in adults or adolescents. However, the skeletal maturation stage is extremely important in cases with tooth loss where space reopening and future rehabilitation with implants are the therapeutic choice. Although relatively important in the posterior region, the vertical growth of the face, which can lead to tooth eruption, in young patients rehabilitated with implants can cause a progressive infraocclusion of the implant. [19]

On average, treatment time in women was 11.2 months longer than in men (*p* = 0.045) in the univariate analysis. However, women had an average of 1.37 missing molars, while for men the average was 0.57. These data corroborate with previous reports that observed a higher frequency of extraction of first molars in women than in men [11]. In multivariate analysis, the tooth loss makes the gender effect insignificant, a confounding variable (*p* = 0.875), confirming data from previous studies in patients without loss. [13, 22, 31]

## Limitations

This is a retrospective study, and therefore, some limitations inherent to this study design must be considered. The sample was collected in a private office, where all patients were treated by the same orthodontist which may be not validated to other operators. All included patients were treated with space closure without TADs, which limits the validation of these results for the treatment model that is probably most used today. Thus, the fact that patients treated with miniscrews were not included may have contributed to a longer treatment time. Furthermore, the results obtained here cannot be extrapolated to orthodontic patients whose planning is space reopening.

Thus, it emphasizes the importance of prospective studies so that these factors can be controlled. Randomized studies comparing groups with loss of the first permanent molar, treated with or without TADs, with reopening or space closure, could elucidate the most efficient way to carry out treatment of patients with first permanent molar loss. Another important aspect to be analyzed would be the effect of space closure on other variables such as root resorption and periodontium.

The high prevalence of patients with loss of the first permanent molar supports the need for studies to assess its influence on treatment time, providing valuable clinical information regarding the predictability of the duration of orthodontic treatment.

## Conclusion

The loss of the first permanent molar causes a longer treatment time in cases in which the remaining space is closed without skeletal anchorage devices. The effect on treatment time is higher the greater the number of tooth losses and the number of arches involved. Treatment time was longer with cases with higher numbers of missed appointments.

## Data Availability

The datasets used and/or analyzed during the current study are available from the corresponding author on reasonable request.

## References

[1] Normando A, Silva M, Le Bihan R, Simone J. Alterações oclusais espontâneas decorrentes da perda dos primeiros molares permanentes inferiores. Rev. dent. press ortodon. ortop. maxilar 2003:15–23.

[2] Normando A, Brandão A, Matos J, Cunha A, Mohry O, Jorge S (1999). Má oclusão e oclusão normal na dentição permanente: um estudo epidemiológico em escolares do município de Belém-PA. Rev Paraense Odontol.

[3] Albadri S, Zaitoun H, McDonnell ST, Davidson LE (2007). Extraction of first permanent molar teeth: results from three dental hospitals. Br Dent J.

[4] Gill DS, Lee RT, Tredwin CJ (2001). Treatment planning for the loss of first permanent molars. Dent Update.

[5] Sabri R. Multidisciplinary management of permanent first molar extractions. Am J Orthod Dentofacial Orthop 2021. 10.1016/j.ajodo.2020.09.024 33495060

[6] Telli A, Aytan S (1989). Changes in the dental arch due to obligatory early extraction of first permanent molars. Turk J Orthodont.

[7] Normando A, Maia F, da Silva Ursi W, Simone J. Dentoalveolar changes after unilateral extractions of mandibular first molars and their influence on third molar development and position. World J Orthodontics 2010;11. 20209178

[8] Normando D, Cavacami C (2010). The influence of bilateral lower first permanent molar loss on dentofacial morfology–a cephalometric study. Dental Press J Orthod.

[9] Saber AM, Altoukhi DH, Horaib MF, El-Housseiny AA, Alamoudi NM, Sabbagh HJ (2018). Consequences of early extraction of compromised first permanent molar: a systematic review. BMC Oral Health.

[10] Abbing A, Koretsi V, Eliades T, Papageorgiou SN (2020). Duration of orthodontic treatment with fixed appliances in adolescents and adults: a systematic review with meta-analysis. Prog Orthod.

[11] Herrera Sanches FS, Santos P, Ferreira MC, Freitas KMS, Henriques JFC, Janson G (2017). Mesiodistal dental movement toward remodeled edentulous alveolar ridge: Digital model assessment. Am J Orthod Dentofacial Orthop.

[12] Serindere G, Bolgul B, Parlar T, Cosgun A (2019). Effects of first permanent molar extraction on space changes observed in the dental arch using data mining method. Niger J Clin Pract.

[13] Fink D, Smith R (1992). The duration of orthodontic treatment. Am J Orthod Dentofacial Orthop.

[14] von Elm E, Altman DG, Egger M, Pocock SJ, Gotzsche PC, Vandenbroucke JP (2008). The Strengthening the Reporting of Observational Studies in Epidemiology (STROBE) statement: guidelines for reporting observational studies. J Clin Epidemiol.

[15] Richmond S, Shaw WC, O'Brien KD, Buchanan IB, Jones R, Stephens CD (1992). The development of the PAR Index (Peer Assessment Rating): reliability and validity. Eur J Orthod.

[16] Ursi WJ, Almeida RR, Tavano O, Henriques JF (1990). Assessment of mesiodistal axial inclination through panoramic radiography. J Clin Orthod.

[17] Tsichlaki A, Chin S, Pandis N, Fleming P (2016). How long does treatment with fixed orthodontic appliances last? A systematic review. Am J Orthodont Dentofac Orthoped.

[18] Papageorgiou SN, Hochli D, Eliades T (2017). Outcomes of comprehensive fixed appliance orthodontic treatment: A systematic review with meta-analysis and methodological overview. Korean J Orthod.

[19] Aghoutan H, Alami S, El Aouame A, El Quars F. Orthodontic management of residual spaces of missing molars: decision factors human teeth-key skills and clinical illustrations. IntechOpen; 2019.

[20] Magkavali-Trikka P, Emmanouilidis G, Papadopoulos M (2018). Mandibular molar uprighting using orthodontic miniscrew implants: a systematic review. Prog Orthod.

[21] Antoszewska-Smith J, Sarul M, Lyczek J, Konopka T, Kawala B (2017). Effectiveness of orthodontic miniscrew implants in anchorage reinforcement during en-masse retraction: a systematic review and meta-analysis. Am J Orthod Dentofacial Orthop.

[22] Melo AC, Carneiro LO, Pontes LF, Cecim RL, de Mattos JN, Normando D (2013). Factors related to orthodontic treatment time in adult patients. Dental Press J Orthod.

[23] Skidmore K, Brook K, Thomson W, Harding W (2006). Factors influencing treatment time in orthodontic patients. Am J Orthodont Dentofac Orthoped.

[24] Richmond S (1993). Personal audit in orthodontics. Br J Orthod.

[25] Mavreas D, Athanasiou AE (2008). Factors affecting the duration of orthodontic treatment: a systematic review. Eur J Orthod.

[26] Dyken R, Sadowsky P, Hurst D (2001). Orthodontic outcomes assessment using the peer assessment rating index. Angle Orthod.

[27] Taylor PJ, Kerr WJ, McColl JH (1996). Factors associated with the standard and duration of orthodontic treatment. Br J Orthod.

[28] Vu CQ, Roberts WE, Hartsfield JK, Ofner S (2008). Treatment complexity index for assessing the relationship of treatment duration and outcomes in a graduate orthodontics clinic. Am J Orthod Dentofacial Orthop.

[29] Robb SI, Sadowsky C, Schneider BJ, BeGole EA (1998). Effectiveness and duration of orthodontic treatment in adults and adolescents. Am J Orthod Dentofacial Orthop.

[30] Stepovich ML (1979). A clinical study on closing edentulous spaces in the mandible. Angle Orthod.

[31] Beckwith FR, Ackerman RJ, Cobb CM, Tira DE (1999). An evaluation of factors affecting duration of orthodontic treatment. Am J Orthod Dentofacial Orthop.

